# In search of a small molecule agonist of the relaxin receptor RXFP1 for the treatment of liver fibrosis

**DOI:** 10.1038/s41598-017-10521-9

**Published:** 2017-09-07

**Authors:** Andrew McBride, Anna M. Hoy, Mark J. Bamford, Danuta E. Mossakowska, Martin P. Ruediger, Jeremy Griggs, Sapna Desai, Kate Simpson, Ivan Caballero-Hernandez, John P. Iredale, Theresa Pell, Rebecca L. Aucott, Duncan S. Holmes, Scott P. Webster, Jonathan A. Fallowfield

**Affiliations:** 1 BHF/University of Edinburgh Centre for Cardiovascular Science, Queen’s Medical Research Institute, 47 Little France Crescent, Edinburgh, EH16 4TJ UK; 2 MRC/University of Edinburgh Centre for Inflammation Research, Queen’s Medical Research Institute, 47 Little France Crescent, Edinburgh, EH16 4TJ UK; 3 Discovery Partnerships with Academia DPU, Gunnels Wood Rd, Stevenage, Hertfordshire, SG1 2NY UK; 4 Platform Technologies and Sciences, GlaxoSmithKline, Gunnels Wood Rd, Stevenage, Hertfordshire, SG1 2NY UK; 5 GlaxoSmithKline, Parque Tecnológico de Madrid, Calle de Severo Ochoa, 2, 28760 Tres Cantos, Madrid Spain

## Abstract

The peptide hormone human relaxin-2 (H2-RLX) has emerged as a potential therapy for cardiovascular and fibrotic diseases, but its short *in vivo* half-life is an obstacle to long-term administration. The discovery of ML290 demonstrated that it is possible to identify small molecule agonists of the cognate G-protein coupled receptor for H2-RLX (relaxin family peptide receptor-1 (RXFP1)). In our efforts to generate a new medicine for liver fibrosis, we sought to identify improved small molecule functional mimetics of H2-RLX with selective, full agonist or positive allosteric modulator activity against RXFP1. First, we confirmed expression of RXFP1 in human diseased liver. We developed a robust cellular cAMP reporter assay of RXFP1 signaling in HEK293 cells transiently expressing RXFP1. A high-throughput screen did not identify further specific agonists or positive allosteric modulators of RXFP1, affirming the low druggability of this receptor. As an alternative approach, we generated novel ML290 analogues and tested their activity in the HEK293-RXFP1 cAMP assay and the human hepatic cell line LX-2. Differences in activity of compounds on cAMP activation compared with changes in expression of fibrotic markers indicate the need to better understand cell- and tissue-specific signaling mechanisms and their disease-relevant phenotypes in order to enable drug discovery.

## Introduction

Fibrotic disorders represent an increasing cause of morbidity and mortality worldwide, contributing to an estimated 45% of all-cause mortality in the United States^1^. In the United Kingdom, liver fibrosis is an extraordinary exception to the major improvements made over the past 30 years in the treatment and outcomes for chronic disorders such as heart disease, stroke and many cancers^2^. Indeed, standardized mortality rates for liver disease have increased inexorably - by 400% since 1970, and in patients younger than 65 years by almost 500%^2^. Despite this significant clinical burden, and major advances in our understanding of the pathogenesis of liver fibrosis, there are still no approved antifibrotic therapies.

Liver fibrosis is the final common pathway of iterative or chronic liver damage^3^. In chronic liver injury, the major profibrogenic cell type is the activated hepatic stellate cell-myofibroblast (HSC-MF), which synthesizes scar tissue and contributes to portal hypertension (PHT) by increasing intra-hepatic vascular resistance through sinusoidal contraction. When fibrosis is advanced, cirrhosis develops characterized by a loss of normal liver architecture, disruption of normal blood flow, the development of nodules of regenerating hepatocytes and consequent functional failure. Cirrhosis is associated with life-threatening complications related to PHT, hepatic failure and the development of hepatocellular carcinoma. The only curative option for end-stage cirrhosis is liver transplantation but donor organ availability cannot meet demand and many patients die waiting for a suitable organ. However, there is now compelling data from both rodent and human models that liver fibrosis is potentially reversible^3^. By studying models of progressive and regressing liver fibrosis it has been possible to identify novel therapeutic targets.

Human relaxin-2 (H2-RLX) is a naturally occurring two-chain peptide hormone of the RLX/insulin peptide family which induces a variety of biological effects in both reproductive and non-reproductive tissues, including modulating cardiovascular and renal physiology as well as mediating anti-inflammatory and antifibrotic effects^4^. H2-RLX circulates in women at low concentrations during the luteal phase of the menstrual cycle and at increased levels during pregnancy, beginning in the first trimester. In men, H2-RLX is detected locally in the prostate and may also be present at very low levels in the circulation. The cognate receptor for H2-RLX, relaxin family peptide receptor-1 (RXFP1), is a member of the leucine-rich repeat (LRR) containing subgroup of G-protein coupled receptors (GPCRs) and is widely distributed in various organs in both sexes^5^. In addition to a seven-transmembrane helix domain, RXFP1 has a large extracellular domain with 10 LRRs and an N-terminal lipoprotein class A domain, which is essential for receptor signaling. Mutagenesis studies have shown that H2-RLX binds with high affinity to the LRRs of the extracellular domain and with lower affinity to the extracellular loops of the transmembrane domain^6–9^.

We have recently demonstrated increased expression of RXFP1 in rat and human HSC-MFs and in a range of experimental models of fibrotic liver disease^10^. Treatment with exogenous H2-RLX attenuated liver fibrogenesis and ameliorated PHT in pathologically distinct rat fibrosis models. In cultured primary human HSC-MFs, H2-RLX inhibited contractility and induced an antifibrogenic phenotype in an RXFP1-dependent manner.

Stimulation of RXFP1 by H2-RLX activates multiple signal transduction pathways including cyclic adenosine monophosphate ((cAMP) influenced by a variety of Gα_s_, Gα_OB_ & Gα_i3_ isoforms), extracellular signal-regulated kinases (ERKs), tyrosine kinases and nitric oxide (NO) signaling, and affects the transcriptional activity of cAMP response element (CRE) and Nuclear factor kappa-light-chain-enhancer of activated B cells (NF-kB) regulated genes^11^. Profibrotic pathways are antagonistically balanced by cAMP, a well-known and conserved antifibrotic second messenger of G-protein coupled signaling cascades^12^. Accumulation of cAMP in HSCs is known to inhibit chemotaxis, proliferation and collagen synthesis, while simultaneously increasing collagen degradation by matrix metalloproteinases (MMPs)^13^. Furthermore, augmentation of intracellular cAMP levels using the cell permeable cAMP analogue dibutyryl cAMP reduced HSC contraction^14^. Mechanistically, cAMP has been shown to inhibit transforming growth factor beta (TGFβ)-SMAD signaling at the transcriptional level *via* modulation of the protein kinase A (PKA)-cAMP response element-binding protein (CREB)-dependent pathway^15^. Although recognized as a key antifibrotic mediator, the mechanism(s) by which cAMP levels are suppressed by multiple receptors during liver fibrogenesis remains unknown^14^.

One of the potential benefits of pharmacologically modulating the H2-RLX/RXFP1 axis in humans has been demonstrated using the investigational drug serelaxin, a recombinant form of H2-RLX, which relieved dyspnoea and reduced mortality in a Phase 3 trial in acute heart failure patients^16^. However, for diseases such as liver fibrosis where chronic administration of H2-RLX would be required, its short *in vivo* half-life and requirement for continuous intravenous or subcutaneous infusion, are among potential obstacles to clinical translation. An alternative approach is therapeutic targeting of RXFP1 using small molecules that act either as agonists or alternatively augment the agonist activity of H2-RLX as positive allosteric modulators (PAMs). A previous study by Xiao and colleagues demonstrated evidence that RXFP1 was chemically tractable^17^, through the identification of a series of RXFP1 agonist compounds following high-throughput screening (HTS) using cAMP as a readout in HEK293T cells stably transfected with RXFP1. Following optimization of initial hits the most promising of these (ML290) was found to stimulate RXFP1 by binding to the extracellular loops of the transmembrane domain and exhibited H2-RLX-like activity by increasing vascular endothelial growth factor (*VEGF*) mRNA expression in a secondary profiling assay using THP-1 cells (human acute monocytic leukemia cell line)^18^. However, the potential role of small molecule RXFP1 agonists/PAMs, including ML290, in treating liver fibrosis has not yet been investigated.

Herein, we strengthen the hypothesis that RXFP1 is a potential therapeutic target for treatment of human liver fibrosis, by showing upregulation of RXFP1 expression in human fibrotic liver tissues. We investigate generation of additional RXFP1 agonists through a cAMP-mediated assay; although high-throughput screening afforded no further specific agonists, we describe the identification of novel, ML290-related activators of RXFP1-mediated cAMP and their use, together with H2-RLX and ML290 in understanding pathways relevant to liver fibrosis, primarily in the hHSC line LX-2.

## Results

### RXFP1 mRNA is expressed in human liver biopsy tissue and localized to areas of fibrotic scarring

**Figure 1:**
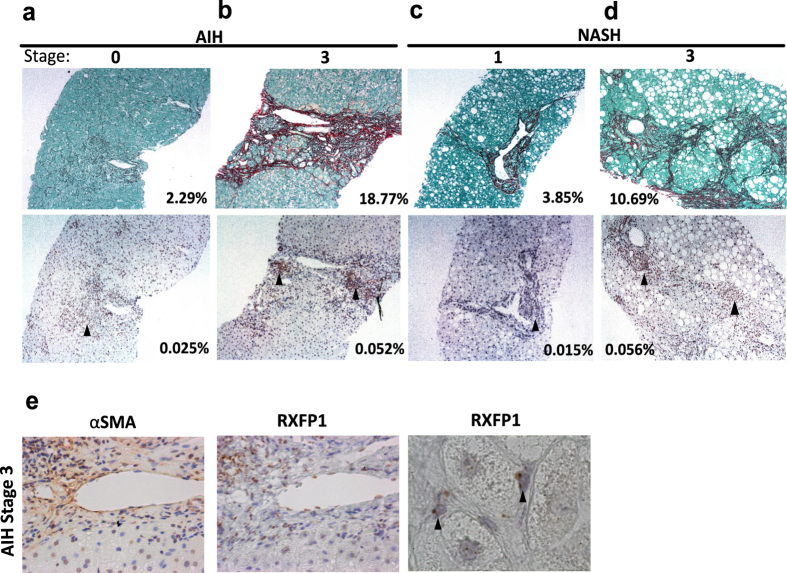
Assessment of fibrosis and *RXFP1* mRNA expression and distribution in human liver biopsies. Representative photomicrographs from illustrative clinical cases of chronic liver disease due to autoimmune hepatitis (AIH) and non-alcoholic steatohepatitis (NASH). Liver fibrosis staged by modified Ishak score (on a 0–6 scale, where 0 = no fibrosis and 6 = cirrhosis). (**a**) AIH Stage 0; (**b**) AIH Stage 3; (**c**) NASH Stage 1; (**d**) NASH Stage 3. Hepatic collagen (red staining) in the biopsy specimens was quantified by morphometry and expressed as collagen proportionate area (%) - top panels; *RXFP1* mRNA expression determined by *in situ* hybridization using oligonucleotide probes designed against human RXFP1 (brown staining, as indicated by the black arrows) – bottom panels. Original magnifications: ×100. (**e**) α-SMA staining of activated HSCs (brown staining; ×400) and RXFP1 mRNA expression determined by *in situ* hybridization (brown staining; ×400 and ×1000, as indicated by the black arrows) of AIH stage 3 liver biopsy specimen.

We have previously shown hepatic expression of RXFP1 after chronic carbon tetrachloride (CCl_4_) and bile duct ligation (BDL) induced liver injury in rats, as well as in explanted human end-stage cirrhotic liver but not in healthy livers^10^. To further explore the clinical relevance of RXFP1 as an antifibrotic target, we examined the expression and distribution of *RXFP1* mRNA by *in situ* hybridization in liver biopsies from patients diagnosed with either early or advanced liver fibrosis due to non-alcoholic steatohepatitis (NASH; n = 2) and autoimmune hepatitis (AIH, n = 2). Expression of *RXFP1* mRNA was detected in the livers of all NASH and AIH patients and localized to areas of fibrotic scarring (identified by picrosirius red stained collagen) (Fig. 1a–d). Cells within the fibrotic scar were found to express α-SMA, an activated hepatic stellate cell marker, with RXFP1 expression localising to morphologically distinctive fibroblast-like cells (Fig. 1e). Furthermore, there was a positive association between liver disease stage and the expression levels of *RXFP1* transcripts.

### Development of a recombinant RXFP1 agonist assay and high-throughput screen

**Figure 2:**
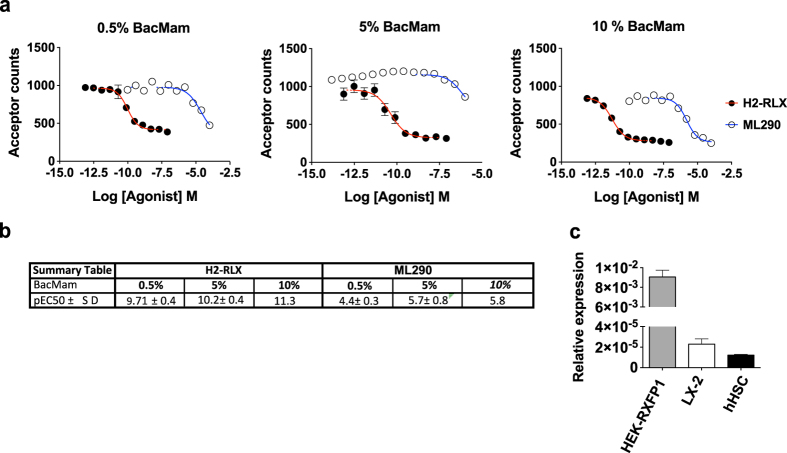
The potency of the H2-RLX and ML290 cAMP response is dependent on the expression level of *RXFP1* mRNA. (**a**) Representative dose-response curves for H2-RLX and ML290 in HEK293 cells transiently transfected with RXFP1 BacMam virus at 0.5%, 5% and 10% v/v. (**b**) Summary table of average H2-RLX and ML290 pEC_50_ values in HEK293 cells transiently transfected with RXFP1 BacMam virus at 0.5%, 5% and 10% v/v. (**c**) Mean relative *RXFP1* transcript level, normalized to beta-actin (*ACTB*) (n = 3), in RXFP1-HEK293 (5% (v/v) BacMam), LX-2 cells and hHSCs p = 0.0002 RXFP1-HEK293 vs LX2 and RXFP1-HEK293 vs hHSC.

In light of the potential clinical relevance of RXFP1 in liver fibrosis we first adopted a HTS approach as a quick and convenient route to potential identification of additional tool molecules for studying RXFP1 signalling in fibrosis-relevant models. The cell of choice in which to screen for receptor agonism would be a relevant human primary cell in which, although in an *in vitro* setting, there would be a greater likelihood of the disease/tissue-relevant receptor coupling mechanism being present. However human-derived primary HSCs are highly plastic and heterogeneous in their behavior^19^ thus making it difficult to screen large numbers of compounds. We therefore developed a robust miniaturized HTS assay using a recombinant system based upon transduction of HEK293 cells with RXFP1 by baculovirus-mediated gene transfer (BacMam)^20^. Stimulation of RXFP1 by H2-RLX in different target tissues activates cAMP signaling^21^. HEK293T-RXFP1 cells have previously been used as a model system for RXFP1-cAMP signalling^22^. Our assay was configured using a Time-Resolved Fluorescence Resonance Energy Transfer (TR-FRET) cAMP immunoassay, initially in 384-well plate format. This assay is based on the competition between a europium-labelled cAMP tracer and cAMP produced by cells, following RXFP1 stimulation, for an Alexa Fluor conjugated cAMP antibody. The resulting FRET signal is inversely proportional to the amount of cellular cAMP generated. The level of *RXFP1* expression varied as a function of the amount of BacMam virus added to the host cells (Fig. 2a,b) and the transient expression of *RXFP1* in HEK293 cells increased the signal window to approximately 3 compared to 1.4 observed in LX-2 cells, where the receptor is endogenously expressed. The determined potency values of H2-RLX, as well as the small molecule RXFP1 agonist ML290, were found to be dependent on the level of *RXFP1* expression; H2-RLX had a mean pEC_50_ of 9.7, 10.2 and 11.3 at 0.5%, 5% and 10% v/v RXFP1-BacMam, respectively. However, even at the highest BacMam concentration tested, the potency of ML290 was significantly lower than previously reported, with a pEC_50_ of 5.8 at 10% v/v BacMam virus compared to the published pEC_50_ of 7.0 reported in HEK293T-RXFP1 and pEC_50_ 6.7 in THP-1 cells^17^. For the HTS assay, 5% v/v BacMam was selected as the optimal transduction level, balancing a sufficient signal window with cell viability. The relative *RXFP1* transcript level in RXFP1 transduced HEK293 cells (5% v/v BacMam) was approximately 400-fold higher than that measured in hHSCs and LX-2 cells that endogenously express RXFP1 (Fig. 2c).

The recombinant RXFP1 assay was configured in PAM mode, where it was run in the presence of a low concentration of H2-RLX (EC_5_). This had the aim of identifying potential PAMs, which augment the effect of H2-RLX, as well as specific RXFP1 agonists. The final assay protocol used for HTS, in 1536-well plates, is described in the methods.

To identify false positives, for example compounds which act on endogenous receptors in HEK293 cells to activate cAMP, hits were counterscreened using a specificity assay comprising the HEK293 cell line without RXFP1-BacMam transduction. The β1/β2 adrenergic receptor agonist isoprenaline was used as a positive control (β1/β2 adrenergic receptors are expressed endogenously in HEK293 cells).

**Figure 3:**
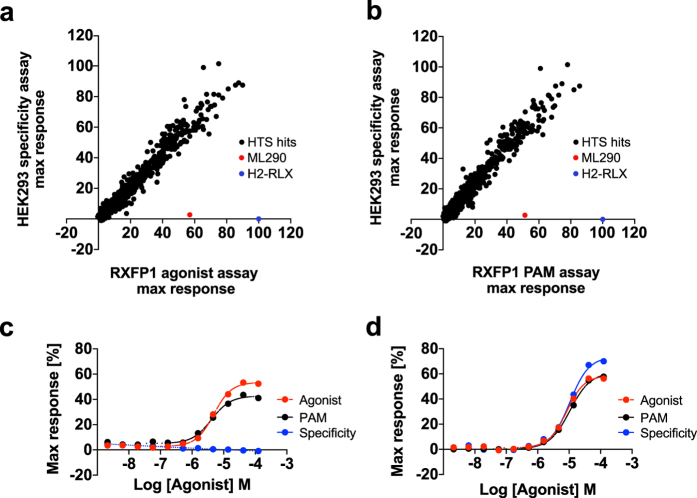
No RXFP1 specific compounds were identified in the HTS. Correlation of the maximum response values observed in dose-response experiments, in the HEK293 host cell specificity assay vs RXFP1 agonist assay (**a**) and vs the RXFP1 PAM assay (**b**). Data are normalised (1% DMSO control = 0%, 100 nM H2-RLX or 10 μM isoprenaline = 100%), and are mean of n = 2. (**c**,**d**): Example dose-response curve data, for ML290 (**c**) and (**a**) non-specific hit compound (**d**). Data are expressed as mean ± SD of n = 6 (**c**) or n = 4 (**d**).

The full GlaxoSmithKline HTS compound library was screened for activity in the RXFP1 PAM assay in single-shot 1536-well plate format at 10 μM compound concentration. The average Z prime (Z’) was 0.79 ± 0.02, with Z’ prime being a measure of assay quality that takes account of both distribution and the difference between the assay high and low controls^23^. Z’ values greater than 0.5 are indicative of a robust screening assay. The average cut-off for agonist activity was 22.9% (calculated as 3 standard deviations, of the inactive population, above the mean of the inactive population). The average signal of the inactive population was centred at 5% response, achieving the target of assaying the compounds in PAM mode. The hit rate was 0.49%, generating an initial list of 8,418 hits, which were expanded to 12,500 compounds (0.79%) by applying plate pattern analysis and region dependent hit rate corrections. 12,250 hits were assayed in confirmation experiments which were run in duplicate for PAM format and for specificity at 10 μM. A comparison of PAM data with the specificity data showed a high degree of correlation, indicating that most compounds were non-specifically increasing cAMP levels in the host cell line. A total of 1,368 compounds were selected for dose-response experiments. However, no compounds were identified which showed a significant window between specific activity on RXFP1 and activity on the host cells (Fig. 3a,b). Representative example curves are shown in (Fig. 3c,d).

### Generation of further ML290-related compounds

Since the screen did not identify additional specific RXFP1 agonists we synthesized novel analogues of the published ML290 structural type, and evaluated their effect on RXFP1 agonist/PAM activity in the RXFP1-HEK293 cell assay. Our series of ML290 analogues had a range of agonist potencies in the HEK293-RXFP1 assay. Indeed, some analogues had no RXFP1 agonist activity whilst others were at least as potent and efficacious in this assay as ML290 (for example compound 2, Table 1). When assessed for specificity in the parental HEK293 cell line ML290 and all of our ML290 analogues had a pEC_50_ less than 3.5.

**Table 1:** RXFP1 agonist activity of ML290 structural analogues.

Compound Name	Chemical Structure	Agonist pEC_50_ (HEK293-RXFP1)	% H2-RLX Maximum Activity
ML290		5.1	55
Compound 1		6.0	64
Compound 2		6.0	104
Compound 3		5.3	38
Compound 4		inactive	inactive

### RXFP1 expression and cAMP signaling in culture-activated primary hHSCs and LX-2 cells

**Figure 4:**
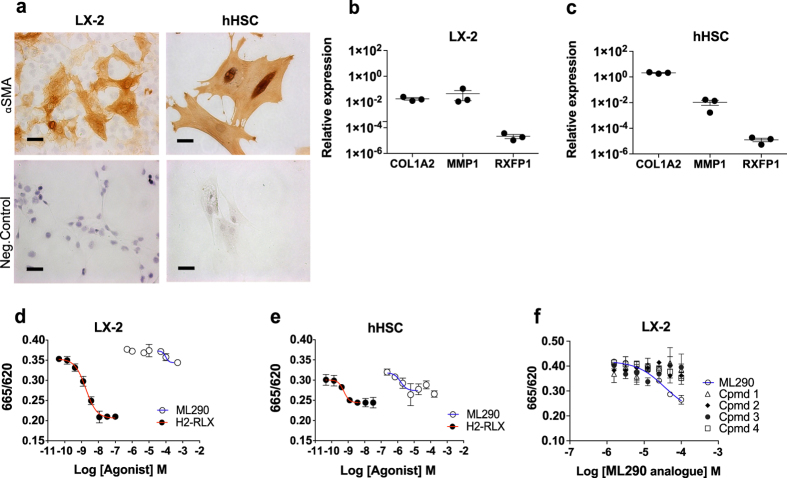
Primary hHSC and LX-2 cells have a profibrogenic phenotype and generate cAMP in response to H2-RLX and ML290. (**a**) Abundant α-SMA staining in hHSCs and LX2 cells indicating an activated myofibroblastic (profibrogenic) phenotype of these cells when cultured on plastic (scale bar = 50 μm). Relative mRNA expression (Ct values) of *ACTB*, *COL1A2*, *MMP1* and *RXFP1* in LX-2 cells (**b**) and hHSCs (**c**); values are the mean ± SD of three independent experiments (LX-2 cells) or three individual patient samples (hHSCs). (**d**) Representative H2-RLX (closed circles) and ML290 (open circles) dose responses in LX-2 cells (pEC_50_ 8.8 and 4.0 respectively) following 40 h of culture on plastic and a 60 min agonist stimulation. (**e**) Representative H2-RLX (closed circles) and ML290 (open circles) dose response in hHSCs (pEC_50_ 9.3, 5.8 respectively) following 40 h of culture on plastic and a 60 min agonist stimulation. (**f**) Representative ML290 analogues dose responses in LX-2 cells following 40 h of culture on plastic and a 60 min agonist stimulation.

In order to explore the relevance of RXFP1-mediated cAMP activation to existing data showing the antifibrotic effects of H2-RLX in fibrotic liver tissues, we went on to explore RXFP1-cAMP signaling responses in more disease relevant cells with endogenous RXFP1 expression. Primary hHSCs and LX-2 cells were cultured in serum-containing media on plastic until a monolayer of alpha-smooth muscle actin (α-SMA) positive myofibroblast-like cells was obtained (Fig. 4a). We showed that both culture-activated hHSC and LX-2 cells endogenously expressed *RXFP1* mRNA by quantitative PCR (qPCR), although relative transcript levels were very low compared to other fibrosis related genes (Fig. 4b,c) and 400-fold lower than *RXFP1* transcript levels in HEK293-RXFP1 cells (Fig. 2b).

LX-2 cells exhibited a cAMP response to a 60 min H2-RLX exposure, following culture for 40 h on plastic to induce an activated fibrotic phenotype (Fig. 4d), with a mean pEC_50_ of 9.1 ± 0.12 (n = 7) and a mean signal to background of 1.4 ± 0.1. The assay was configured in 96-well plates with an optimum seeding density of 10,000 cells/well. Next, we showed that primary hHSCs elicited a cAMP response to a 60 min exposure to H2-RLX following a 40 h culture in 96-well plates (10,000 cells/well) to establish an activated HSC-MF phenotype (Fig. 4e). Notably, the cAMP response to H2-RLX in hHSCs was similar to that observed in LX-2 cells with a mean pEC_50_ across three patient samples of 9.0 ± 0.13, with a mean signal to background of 1.5 ± 0.1.

We also tested the ability of ML290^17^ and related compounds to stimulate a cAMP response in both the LX-2 and hHSC assay formats (Fig. 4d,e). The cAMP response of LX-2 cells to ML290 was much lower in magnitude (20% of the response to H2-RLX) and potency (pEC_50_ 4.5 ± 0.54, n = 6) (Fig. 4d,e) and the ML290 related compounds (Table 1) did not show any significant cAMP-mediated agonist activity (Fig. 4f). Interestingly, ML290 was found to be more potent in the hHSC assay with a pEC_50_ of 5.33 ± 0.89, n = 4.

Thus, the potency and efficacy of ML290 as an RXFP1 agonist was lower in our HEK293-RXFP1, LX-2 and hHSC assays than previously reported for this compound in HEK293T cells stably transfected with RXFP1^17^.

### Assessment of RXFP1 mediated antifibrotic activity in LX-2 cells

**Figure 5:**
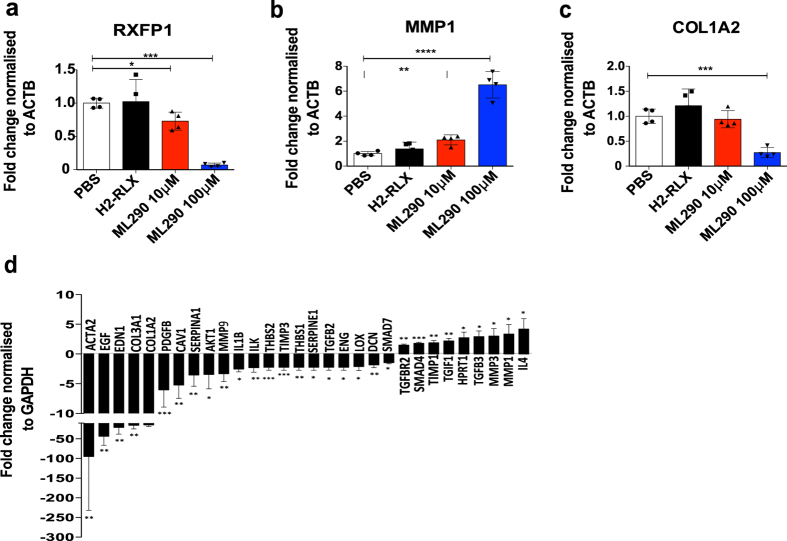
Compound ML290 induced modulation of fibrosis related gene transcripts in LX-2 cell line. LX-2 cells were treated with 10  μM or 100 nM of ML290, 100 nM H2-RLX or PBS (control) for 24 h (n = 4). Expression of (**a**) *RXFP1*, (**b**) *MMP1*, (**c**) *COL1A2* mRNA was quantified by qPCR, normalized to beta-actin (*ACTB*) and fold changes were calculated as a ratio of values from treated to PBS controls. (**d**) Investigation of antifibrotic effects using qPCR array to quantify fibrosis related gene transcripts in LX-2 cells treated with 100 µM ML290. mRNA expression was normalized to *GAPDH* and fold changes were calculated as a ratio of values from treated to PBS controls. (NS not significant, *p < 0.05, **p < 0.01, ***p < 0.001, ****p < 0.0001).

Downstream signaling events in HSCs (and other tissue myofibroblasts) in response to H2-RLX include transcriptional modulation of genes implicated in fibrogenesis (e.g. α-SMA, type-I collagen, tissue inhibitor of metalloproteinase-1 (TIMP-1)) and fibrolysis (e.g. MMPs)^10, 24, 25^. To demonstrate proof of concept that a synthetic RXFP1 agonist could induce a detectable antifibrotic signal *in vitro* we studied the effect of H2-RLX and compound ML290 on gene transcription in LX-2 cells, at concentrations that induced a maximal cAMP response. We showed that the expression of hepatic fibrosis marker genes in LX-2 cells was modulated in a dose-dependent manner following 24 h treatment with ML290, but not H2-RLX (Fig. 5a,c). Notably, whilst the expression of type-I collagen (*COL1A2*) transcripts was reduced 3.3 ± 0.5–fold (n = 2), *MMP1* expression was increased 8.0 ± 2.0–fold (n = 2) in response to ML290. We have previously shown that RXFP1 expression is a marker of the activated hHSC-MF phenotype, whereas hHSC-MFs that were deactivated by re-plating onto basement membrane matrix (Matrigel) down-regulated expression of RXFP1^10^. Following treatment with 100 μM ML290, but not 10 μM ML290, the level of RXFP1 transcripts in LX-2 cells was reduced 9.8 ± 6.4 –fold (n = 2). In contrast, no significant effect on gene transcription was observed in response to H2-RLX at 100 nM under the same conditions. A more extensive evaluation of the gene modulatory effects of ML290 in LX-2 cells was performed using a human fibrosis qPCR array (Fig. 5d). The qPCR array confirmed broad antifibrotic activity including, in particular, significant down-regulation of HSC activation markers (α-SMA (*ACTA2*), 95-fold reduction (p = 0.003); platelet derived growth factor B (*PDGFB*), 6-fold reduction (p = 0.0003)) and fibrillar collagens (*COL1A2*, 14.6-fold reduction (p = 0.067); type-III collagen (*COL3A1*), 15.6-fold reduction (p = 0.007)), and upregulation of *MMP1* (3.3-fold increase (p = 0.028)). The fibrogenic gene modulatory effects of H2-RLX and ML290 on fibrotic marker genes was further assessed in hHSCs, however the effects were variable and not sufficiently reproducible to permit a detailed analysis.

## Discussion

Chronic liver disease (CLD) is the only major cause of death that is currently increasing year on year, representing a significant and growing healthcare concern^1^. As no approved antifibrotic therapies are currently available, the development of new medicines to halt or reverse fibrosis are urgently needed. We and others have identified that the mammalian peptide hormone H2-RLX has potential as an antifibrotic treatment^10, 26–29^. The major cellular targets of H2-RLX in the liver are activated HSCs, which are the primary source of extracellular matrix (ECM) constituents in liver fibrosis. Activated HSCs upregulate expression of the RLX receptor, RXFP1, whereas RXFP1 is undetectable in quiescent HSCs^10, 30^.

Here we showed that *RXFP1* mRNA localized to regions of scarring in the livers of patients with aetiologically distinct fibrotic liver diseases. The association between *RXFP1* expression and liver fibrogenesis was reinforced by the positive correlation between *RXFP1* transcript level and stage of fibrosis. We hypothesized that agonism of RXFP1 in HSCs would ameliorate fibrosis in patients with CLD. Recent clinical reports showing that RXFP1 is downregulated in lung tissue in idiopathic pulmonary fibrosis and in skin lesions in scleroderma highlights the importance of understanding the expression and regulation of RXFP1 in different tissues in health and disease^31, 32^.

Finding small molecule agonists of large N-terminal domain Family A GPCRs such as RXFP1 can be challenging, although there are examples of receptors in this class for which ligands have been identified^33^. Current HTS technologies offer a convenient and rapid mechanism to explore very large compound libraries to identify starting points for small molecule drug discovery or tools for further investigation of biology. Adopting this approach, we conducted a HTS against the GlaxoSmithKline (GSK) compound collection. Our primary assay utilized cAMP as a robust measure of receptor activity in HEK293 cells transiently expressing human RXFP1. Although there are potential alternative RXFP1 signaling pathways that could be interrogated as a measure of receptor activity^34^, other investigators had previously used the cAMP approach to identify small molecule RXFP1 agonists^17, 18, 35^. Despite developing a robust, sensitive and validated screening assay we failed to identify additional RXFP1 agonists by this approach, further suggesting that this receptor is poorly tractable to existing small molecule drug discovery approaches.

In order to identify a broader selection of tool molecules with which to further investigate RXFP1 mediated signaling and biology, we therefore explored additional analogues of ML290, the only RXFP1 agonist chemotype reported to date. Our novel ligands included those shown in Table 1. Examples were identified which showed pEC_50_ agonist potencies in the recombinant RXFP1-expressing HEK293 cAMP assay as high as that for ML290, with efficacies also at least as high, including example 2 (Table 1) with 100% efficacy (versus H2-RLX) in this assay.

With tool compounds in-hand, we set out to investigate RXFP1 mediated signaling and gene expression in our recombinant and human disease tissue-relevant cells. We compared RLX dependent cAMP signaling in primary hHSC and LX-2 cells with that of HEK293-RXFP1 cells. Although all three cell types were found to express RXFP1, the endogenous levels of *RXFP1* mRNA in hHSC and LX-2 cells were 400-fold lower than those found in HEK293-RXFP1 cells (Fig. 2b) used in our HTS. Indeed, the level of *RXFP1* expression in HEK293 cells, and consequently RLX dependent cAMP generation, could be readily adjusted in HEK293 cells using the BacMam system. This had the advantage of allowing the signal window to be increased from 1.4 in LX-2 and hHSCs to greater than 3 in the HEK293-RXFP1 cells. The artificially high RXFP1 expression in HEK293 cells also had the effect of increasing the potency of the cAMP response of RLX from a pEC_50_ of 9.0 in LX-2 and HSCs to a pEC_50_ of 11 in HEK293-RXFP1 cells. While our primary assay has the potential to exaggerate agonist potency, secondary assays in LX-2 or HSC assays would have utility in confirming agonist activity.

We also investigated the cAMP response of the known RXFP1 agonist ML290 in each cell type. While ML290 elicited a cAMP response in all three cells, it showed a different magnitude of effect and potency between the different cell types, which might be due to cell-specific differences in the complement and expression of G proteins (G_αs_, G_αoB_ or G_αi3_) that couple to RXFP1^21^. Interestingly, the potency of ML290 was found to be lower in LX-2 cells (pEC_50_ 4.0) than in hHSCs (pEC_50_ 5.8), despite similar cAMP responses to H2-RLX in these cells. In our HEK293-RXFP1 cells ML290 induced a 3-fold increase in intracellular cAMP with a pEC_50_ of 5.1, compared to the reported pEC_50_ of 7.0 in HEK293T-RXFP1^17^. The reduced potency of ML290 in our cell assays may reflect differences in RXFP1 expression and assay conditions from that used by Xiao and colleagues^17^. Furthermore, none of the novel ML290-related compounds showed an effect on cAMP accumulation in LX2 cells. Together, these disparities between the H2-RLX and ML290 chemotype cAMP responses suggest both cell-dependent differences in downstream signaling following agonist exposure and differences in the distinct mechanisms by which ML290, its analogues and H2-RLX engage and activate RXFP1. This is perhaps not surprising as ML290 interacts with human RXFP1 at a site distinct from that of H2-RLX, with the low density lipoprotein class A (LDLa) domain of RXFP1 being dispensable for activity^17^. Furthermore, the actions of ML290 are species-specific, with activity at human, macaque and pig RXFP1 but no agonist action at the mouse, rat, hamster or guinea pig receptor^36^. Amino acid differences in the third extracellular loop of the seven-transmembrane region of RXFP1 are responsible for this specificity. Such mechanistic differences in receptor engagement and activation could translate into divergent activation of downstream signaling pathways. These alternative pathways include NO/NO synthase (NOS)/ cyclic guanosine monophosphate (cGMP), NFκB, PKA, ERK1/2 and phosphoinositide 3-kinase (PI3K)^34, 35, 37, 38^. In support of this it has been reported that a peptide analogue of the B chain of H2-RLX preferentially activates the pERK pathway over cAMP signaling to promote antifibrotic cell responses in human cardiac fibroblasts and rat renal myofibroblasts^39^. This underlines the complexity of the RXFP1 signaling axis and raises the possibility that cAMP may not necessarily be the best indicator of RXFP1 mediated antifibrotic activity.

Therefore, we sought to determine the correlation between RXFP1-mediated induction of cAMP signaling and downstream, disease-relevant effects by profiling changes in fibrotic gene expression in LX-2 cells treated with ML290. ML290 induced a dose-dependent increase in *MMP1* transcripts, with a reduction in procollagen (*Col1a2*) and *RXFP1* transcripts. The most striking antifibrotic response was at a dose of 100 μM, which correlated with the maximal cAMP response to ML290 in these cells. This antifibrotic gene expression profile was validated in a real-time PCR array, where ML290 down-regulated α-SMA and fibrillar collagens, whilst up-regulating several proteases (including *MMP1*). Surprisingly, H2-RLX did not induce a similarly reproducible antifibrotic signature under the same assay conditions at the H2-RLX concentration which resulted in maximal cAMP activation. This may reflect the fact that LX-2 cells had the smallest and least potent H2-RLX dependent cAMP response in our assays, being of insufficient amplitude to be translated into downstream gene expression changes. Furthermore, although others have previously shown the antifibrotic effect of 1 nM to 1 μM H2-RLX in primary rat and human HSCs *in vitro*, it is also possible that the maximal cAMP response concentration of H2-RLX (100 nM) does not achieve the cellular threshold concentration to elicit antifibrotic effects in LX2 cells. This result brings into question the suitability of cAMP accumulation as the optimal measure of RXFP1 mediated antifibrotic signaling activity. Despite endogenous expression of RXFP1, LX-2 cells exhibit a complex karyotype and cellular heterogeneity^40^. Indeed, not all cells expressing RXFP1 have robust RLX-dependent cAMP responses^25, 41, 42^. Alternatively, ML290 may be inducing antifibrotic gene changes that are independent of RXFP1, especially given the high concentration of compound required in these experiments.

Despite the obvious advantages of an orally-active small molecule functional mimetic over the recombinant H2-RLX peptide as a chronic therapy for fibrosis, the discovery of novel RXFP1 agonist compounds will remain a significant challenge without a deeper understanding of tissue/cell specific differences in RXFP1 expression, signaling and its regulation of dependent genes in health and disease. Such knowledge will enable the development of optimized screening cascades and functional assays using therapeutically relevant cells, expressing physiological densities of RXFP1.

## Methods

Human tissue was used in accordance with the Human Tissue (Scotland) Act 2006. Prospective human liver samples were collected after informed consent under the framework of the Lothian NRS Human Annotated Bioresource (Research Ethics Committee reference: 10/S1402/33). The study was approved by the NHS Lothian Tissue Governance Committee.

### Cell isolation and culture

Primary HSCs were isolated from normal human liver samples (prospectively collected from living donors and consisting of residual tissue surplus to diagnostic requirements) using pronase/collagenase digestion and density gradient centrifugation, and cultured on plastic in Dulbecco’s modified Eagle’s medium (DMEM; Gibco, Life Technologies, UK) with 16% fetal bovine serum (FBS) supplemented with antibiotics for 10-14 days at 37 °C/5% CO_2_ until a monolayer of myofibroblast-like activated HSCs was obtained. The immortalized HSC line LX-2 were a gift from Dr Scott Friedman (Mt Sinai, USA) and were cultured in DMEM with 10% FBS supplemented with antibiotics at 37 °C/5% CO_2_.

### cAMP assay

cAMP generation in LX-2 and hHSC cells was measured using the Lance cAMP kit (Perkin Elmer, UK) according to the manufacturer’s instructions. Cells were plated on 96-well plates at a density of 10,000 cells/well and cultured for a minimum of 40 h at 37 °C/5%CO_2_, prior to a 60 min agonist incubation in Hanks Balanced Salt Solution (HBSS), 5 mM 4-(2-hydroxyethyl)−1-piperazineethanesulfonic acid (HEPES) pH 7.4, supplemented with 0.01% bovine serum albumin (BSA), and 0.5 mM isobutylmethylxanthine (IBMX). Lance Antibody detection reagent was then added and the Fluorescence Resonance Energy Transfer (FRET) signal read 4 h later on a Tecan Infinite M1000 plate reader (Tecan UK). FRET response was expressed as ratiometric value of the 665 nm and 620 nm fluorescence signals.

### *In situ* hybridisation

*RXFP1* mRNA *in situ* hybridization was performed using RNAscope 2.0 HD Brown Reagent Kit (Advanced Cell Diagnostics, Hayward, CA) and paired double-Z oligonucleotide probes designed against human RXFP1 (NM_001253730.1, 20 “‘zz” oligo pairs, probe region: 476-1721) according the manufacturer’s protocol. Briefly, 4% normal buffered formalin-fixed, paraffin-embedded human liver biopsy specimens were sectioned at 5 μm Tissues were deparaffinized, dehydrated and treated with peroxidase block for 10 min at room temperature prior to boiling in a pretreatment solution for 15 min and proteinase K treatment for 30 min at 40 °C. RXFP1 probe, and control positive and negative probes, cyclophilin B (PPIB) and bacterial gene dapB respectively were hybridized for 2 h at 40 °C, followed by a series of signal amplification and washing steps. The signal visualization was performed using diaminobenzidine (DAB) solution for 10 min at RT and the sections were counterstained for 2 min with Gill’s Hematoxylin.

### Fibrosis staging, picrosirius red and αSMA staining

Human liver biopsies were staged for fibrosis by modified Ishak score (MIS) (scale 0–6) and collagen proportionate area (CPA, %) was calculated morphometrically using ImageJ^43^ (National Institutes of Health, USA) after picrosirius red staining, as previously described^44^. α-SMA staining was performed as described previously^10^.

### Quantitative RT-PCR (including qPCR array)

LX-2 or HSC cells were seeded overnight on 24-well plates at a density of 50,000 cells per well. Cell media was then replaced with fresh media supplemented with RXFP1 agonist and incubated for a further 24 h at 37 °C/5% CO_2_. RNA was extracted from cell lysates using the RNeasy Kit (Qiagen, UK), and reverse transcribed to cDNA using the QuantiTect Kit (Qiagen, UK), according to manufacturer’s instructions. qPCR analysis of cDNA was performed using a LightCycler 480 qPCR machine and primer and probe sets designed against target genes using the Roche Universal ProbeLibrary Assay Design Centre (https://lifescience.roche.com/webapp). Primer details can be found in supplementary information. Raw threshold values (Ct) were normalized to beta-actin (*ACTB*) and fold change calculated relative to untreated controls using the 2^−ΔΔC^t method. The Qiagen RT^2^ Profiler PCR Human Fibrosis Array (Qiagen, UK) was used according to the manufacturer’s instructions. Raw threshold values (Ct) were normalized to *GAPDH* and fold change calculated relative to untreated controls using the 2^−ΔΔC^t method.

### Bacmam-HEK293 (TR-FRET cAMP) HTS assay

Recombinant RXFP1 baculoviruses (Bacmam) was generated according to manufacturer’s instructions using the Bac-to-Bac system (Invitrogen, UK). HEK293 (ATCC, USA) cells were transiently transduced with 5% (v/v) RXFP1 BacMam to generate vials of frozen, assay-ready cells in sufficient quantities to support HTS. cAMP generation in RXFP1-HEK293 cells was measured using the Lance cAMP kit (Perkin Elmer, UK) according to the manufacturer’s instructions. Frozen RXFP1-HEK293 cells were defrosted and resuspended in assay buffer (HBSS, 5 mM Hepes pH 7.4, 0.01% BSA, and 0.5 mM IBMX). The cell solution was plated at a density of 2,000 cells/well in 2 μl into 1536-well plates pre-stamped with 50 nl of test compound in 100% DMSO. Lance stimulation reagent, supplemented with 5 pM Relaxin (Bachem AG, Switzerland) for positive modulator-mode assays, was then added at 2 μl/well. After a 60 min agonist incubation 4 μl/well Lance detection reagent was added and the FRET signal read 2 h later on a Viewlux plate reader (Perkin Elmer, UK). Results were expressed as acceptor fluorescence (665 nm) normalised to a Relaxin high control (100 nM). Assays post-HTS were performed in 384-well format, using 10,000 cells/well in 10 μl into plates pre-stamped with 100 nl of test compounds in 100% DMSO. Detection was enabled with 10 μl of Lance stimulation and 20 μl of detection buffer.

### Specificity assay

As per the HTS assay, utilizing HEK293 cells not transfected with RXFP1 or stimulated with H2-RLX. Results were expressed as acceptor fluorescence (665 nm) normalised to an Isoprenaline (Sigma, UK) high control (10 µM).

### Synthesis and analysis of ML290 and ML290 analogues

ML290 was purchased from Merck Chemicals (Cat. No. 505876) with a purity of 99.3%. ML290 analogues, Compounds 1 to 4 were synthesized by BioDuro, with a purity ≥96%.

### Statistical analysis

The results were given as mean ± SD from two or seven independent experiments. The statistical significance of results was determined using a two-tailed unpaired Student’s t-test. p value less than 0.05 (p < 0.05) was considered statistically significant.
